# Supplementary material for: Bilateral human laryngeal motor cortex in perceptual decision of lexical tone and voicing of consonant
Source: Nat Commun. 2023 Aug 5;14:4710. doi: 10.1038/s41467-023-40445-0 (PMC10404239; doi:10.1038/s41467-023-40445-0)
Supplement: Supplementary file 3 — Description of Additional Supplementary Files [file 41467_2023_40445_MOESM3_ESM.pdf]

Description of Additional Supplementary Files

File name: Supplementary Audio 1

Description: Clear syllables [ti55], [ti35], [t^hi55], and [t^hi35] for continua synthesis.

File name: Supplementary Audio 2

Description: Morphed five-step lexical tone (F0) continuum from [ti55] to [ti35].

File name: Supplementary Audio 3

Description: Morphed five-step consonant (VOT) continuum from [ti55] to [t^hi55].

Supplementary Audio 2–3 were selected from the individualized stimuli matrix from one participant in Experiment 1.
