# Supplementary material for: Bilateral human laryngeal motor cortex in perceptual decision of lexical tone and voicing of consonant
Source: Nat Commun. 2023 Aug 5;14:4710. doi: 10.1038/s41467-023-40445-0 (PMC10404239; doi:10.1038/s41467-023-40445-0)
Supplement: Supplementary file 7 — Reporting Summary [file 41467_2023_40445_MOESM7_ESM.pdf]

nature portfolio

Corresponding author(s): Yi Du

Last updated by author(s): Jul 15, 2023

Reporting Summary

Nature Portfolio wishes to improve the reproducibility of the work that we publish. This form provides structure for consistency and transparency in reporting. For further information on Nature Portfolio policies, see our Editorial Policies and the Editorial Policy Checklist.

Statistics

For all statistical analyses, confirm that the following items are present in the figure legend, table legend, main text, or Methods section.

n/a	Confirmed	
☐	☒	The exact sample size (n) for each experimental group/condition, given as a discrete number and unit of measurement
☐	☒	A statement on whether measurements were taken from distinct samples or whether the same sample was measured repeatedly
☐	☒	The statistical test(s) used AND whether they are one- or two-sided<br>*Only common tests should be described solely by name; describe more complex techniques in the Methods section.*
☒	☐	A description of all covariates tested
☐	☒	A description of any assumptions or corrections, such as tests of normality and adjustment for multiple comparisons
☐	☒	A full description of the statistical parameters including central tendency (e.g. means) or other basic estimates (e.g. regression coefficient) AND variation (e.g. standard deviation) or associated estimates of uncertainty (e.g. confidence intervals)
☐	☒	For null hypothesis testing, the test statistic (e.g. F, t, r) with confidence intervals, effect sizes, degrees of freedom and P value noted<br>*Give P values as exact values whenever suitable.*
☒	☐	For Bayesian analysis, information on the choice of priors and Markov chain Monte Carlo settings
☐	☒	For hierarchical and complex designs, identification of the appropriate level for tests and full reporting of outcomes
☐	☒	Estimates of effect sizes (e.g. Cohen's d, Pearson's r), indicating how they were calculated

Our web collection on statistics for biologists contains articles on many of the points above.

Software and code

Policy information about availability of computer code

Data collection	Sample sizes were estimated before experiments started using G*Power 3.1.<br>For auditory stimuli generation, we used Legacy-STRAIGHT (2018, latest version) in Matlab R2016a platform and praat 6 to re-synthesize the syllables.<br>For the functional localization test, we used Matlab R2016a and Psychtoolbox-3 to present visual stimuli.<br>For TMS stimulation, we used Brainsight 2.4 neuronavigation software to navigate the coil.<br>For the behavioral data collection, we used Matlab R2016a and Psychtoolbox-3 to present auditory stimuli.
Data analysis	For the processing of neuroimaging data, we used MRIcron (2016) to process T1 images, fMRIprep (20.2.5) to preprocess functional images (also recruiting packages from ANTs 2.3.3, FSL 5.0.9, AFNI 20160207, and AFNI 20220424), and AFNI 20220424 for subject and group level GLM analyses. Visualization of neuroimaging data and schematic illustration were implemented by Mango 4.1, BrainNet Viewer 1.7, and Matplotlib based on Python 2.7.<br><br>For the psychometric curve fitting for behavioral data, we used customized Matlab code (https://zenodo.org/record/8075062).<br>For the drift-diffusion modeling (DDM) for behavioral data, we used hierarchical Bayesian estimation of DDM using the HDDM (0.9.8) package based on Python 2.7.<br>For the reaction time analyses for behavioral data, we used lmer4 and lmerTest packages in Rstudio 1.4.1103 (R, 4.0.4) to build linear mixed models.<br>Visualization of behavioral data was used by Matlab R2021a, Microsoft PowerPoint 2019, and Adobe Illustrator 2020.

For manuscripts utilizing custom algorithms or software that are central to the research but not yet described in published literature, software must be made available to editors and reviewers. We strongly encourage code deposition in a community repository (e.g. GitHub). See the Nature Portfolio guidelines for submitting code & software for further information.

Data

Policy information about availability of data

All manuscripts must include a data availability statement. This statement should provide the following information, where applicable:

- Accession codes, unique identifiers, or web links for publicly available datasets
- A description of any restrictions on data availability
- For clinical datasets or third party data, please ensure that the statement adheres to our policy

We provided raw data in Experiment 1 and 2, as well as the bold activation maps for the fMRI pretest. Raw data and instructions for usage have been deposited in the Zenodo database (https://zenodo.org/record/8092010). Source data are provided with this paper.

Research involving human participants, their data, or biological material

Policy information about studies with human participants or human data. See also policy information about sex, gender (identity/presentation), and sexual orientation and race, ethnicity and racism.

Reporting on sex and gender	We collected the participants' gender on resident identity card that usually but not necessarily represent their assigned gender and/or gender identity. Nevertheless, we did not perform any aggregation by gender and do not expect gender differences in low-level speech perception.
Reporting on race, ethnicity, or other socially relevant groupings	Participants were graduate students or employees in Beijing with higher education. We did not expect the social groupings would affect our results as we tested basic auditory neural processing and speech perception.
Population characteristics	Functional localization experiment: 48 right-handed adults (26 females, mean age = 21.44, SD = 2.79).<br>Experiment 1: 64 right-handed adults (36 females, mean age = 22.19 years, SD = 2.88). One left before finishing sham and dLMC stimulation condition, leaving 63 participants with valid data.<br>Experiment 2: 26 right-handed adults (14 females, Mean age = 22.38 years, SD = 2.88). One stopped after one session with iTBS upon the right dLMC, leaving 25 participants with valid data.<br>Supplementary Experiment 1: 22 right-handed adults (13 females, mean age 21.32 years, SD = 2.16).<br>Supplementary Experiment 2: 58 right-handed adults (29 females, mean age = 21.31 years, SD = 2.93).<br>All participants had normal hearing. None of them had self or family history of neurological, traumatic, or psychiatric diseases. Participants were Mandarin Chinese speakers, and were all non-musicians.<br>Overlap and repeat measures of participants in different experiments are reported in the manuscript.
Recruitment	Participants were students or employees in Beijing with higher education, and were recruited through advertisement (convenience sampling). Most participants were undergraduate students, graduate students, and researchers at the institute or nearby universities. It is unlikely that the study of low-level sensory processing would be biased by the selection procedure.
Ethics oversight	The experiment was approved by the Ethics Committee of the Institute of Psychology, Chinese Academy of Sciences.

Note that full information on the approval of the study protocol must also be provided in the manuscript.

Field-specific reporting

Please select the one below that is the best fit for your research. If you are not sure, read the appropriate sections before making your selection.

☐ Life sciences ☒ Behavioural & social sciences ☐ Ecological, evolutionary & environmental sciences

For a reference copy of the document with all sections, see nature.com/documents/nr-reporting-summary-flat.pdf

Behavioural & social sciences study design

All studies must disclose on these points even when the disclosure is negative.

Study description	This study is a quantitative experimental design combing non-invasive brain stimulation and behavioral testing with model fitting.
Research sample	Functional localization experiment: 48 right-handed adults (26 females, mean age = 21.44, SD = 2.79).<br>Experiment 1: 64 right-handed adults (36 females, mean age = 22.19 years, SD = 2.88). One left before finishing sham and dLMC stimulation condition, leaving 63 participants with valid data.<br>Experiment 2: 26 right-handed adults (14 females, Mean age = 22.38 years, SD = 2.88). One stopped after one session with iTBS upon the right dLMC, leaving 25 participants with valid data.<br>Supplementary Experiment 1: 22 right-handed adults (13 females, mean age 21.32 years, SD = 2.16).<br>Supplementary Experiment 2: 58 right-handed adults (29 females, mean age = 21.31 years, SD = 2.93).<br>All participants had normal hearing. None of them had self or family history of neurological, traumatic, or psychiatric diseases.<br>Participants were Mandarin Chinese speakers, and were all non-musicians.

	Overlap and repeat measures of participants in different experiments are reported in the manuscript.<br>Most participants were undergraduate students, graduate students, and researchers at the institute or nearby universities. We believe that the selected sample can represent the young healthy adult population in terms of the targeted cognitive neural mechanism as it is unlikely that the study of low-level sensory processing would be biased by the selection procedure.
Sampling strategy	Participants were students or employees in Beijing with higher education, and were recruited through advertisement (convenience sampling). Different groups of participants for group comparisons were matched in age, sensitivity to stimulation, and handedness. Sample sizes are sufficient to detect medium to large effects as estimated by G*Power 3.1.
Data collection	Functional localization experiment: participants needed to articulate the voiceless [t] (only tongue moved) or pronounce the voiced [a] (only laryngeal vocal fold vibrated) in the MRI scanner. No other persons were in the room with the participant. The experimenter B.S.L. operated the presentation program outside the room and knew the goals as well as the ongoing conditions of the experiment. TMS experiments: participants performed syllable identification tasks during (Experiment 1) or after (Experiment 2) receiving TMS stimulation. Biphasic magnetic pulses were generated by a standard figure-of-eight coil from a Magstim Rapid2 stimulator. Stimuli were played by a Sennheiser in-ear headphones (IE 60) connected to a Steinberg UR 242 amplifier. Before and during TMS application, experimenters B.S.L. and Y.C.L. set up the protocols, applied the stimulation, and ran the experiment program. In Experiment 1, during the tasks, B.S.L. ran the presentation program while Y.C.L. applied stimulation. In Experiment 2, during the tasks, one experimenter ran the program while the other left the room. This is a single-blinded design where participants were not told whether they accepted real or sham stimulation. However, experimenters were not blinded since they had to monitor the coil, but it is unlikely that experimenters' states would affect the observation of participants' low-level perception decision.
Timing	Functional localization experiment: Oct. 9, 2019 to Nov. 4, 2019.<br>Experiment 1: Oct. 30, 2020 to Nov. 20, 2020; Jan. 19 to Jan. 29, 2021. Two periods shared no participants, and it is unlikely that seasons affected our observations.<br>Experiment 2: Mar. 31 to Jun. 29, 2021.<br>Supplementary Experiment 1: Jul. 1 to Jul. 30, 2019.<br>Supplementary Experiment 2: Oct. 9, 2019 to Nov. 4, 2019.
Data exclusions	For psychometric curve fitting, slopes with negative values (< 0) or with the point of subjective equity out of the continuum interval (< 1 or > 5) were eliminated as these parameters were mathematically invalid in the current context. For TMS conditions, the invalid slopes were replaced by the floor values from the corresponding sham conditions. Number of eliminated invalid slopes removed as invalid data and replaced by sham floor values are shown in Supplementary Table 2. Results without such a replacement procedure for Experiment 2 were also provided in Supplementary Fig. 2.<br>No specific data exclusion criteria were applied in the drift-diffusion modeling of and reaction time analyses of the behavioral data, and processing of neuroimaging data.
Non-participation	One participant in Experiment 1 and another one participant in Experiment 2 quit because they were not willing to accept brain stimulation. In addition, for Experiment1, in each group, 2 participants did not finish the tongue motor cortex stimulation sessions. All participants gave written informed consent prior to the experiment and had told that they could quit the experiment at any time for no reason.
Randomization	Different groups of participants for group comparisons were matched in age, sensitivity to stimulation, and handedness.<br>For all experiments, the order of sessions and blocks was randomized using Latin square procedure. Trials were presented randomly in behavioral experiments.<br>The order of keys for the response was balanced across participants.

Reporting for specific materials, systems and methods

We require information from authors about some types of materials, experimental systems and methods used in many studies. Here, indicate whether each material, system or method listed is relevant to your study. If you are not sure if a list item applies to your research, read the appropriate section before selecting a response.

Materials & experimental systems

n/a	Involved in the study
☒	☐ Antibodies
☒	☐ Eukaryotic cell lines
☒	☐ Palaeontology and archaeology
☒	☐ Animals and other organisms
☒	☐ Clinical data
☒	☐ Dual use research of concern
☒	☐ Plants

Methods

n/a	Involved in the study
☒	☐ ChIP-seq
☒	☐ Flow cytometry
☐	☒ MRI-based neuroimaging

Magnetic resonance imaging

Experimental design

Design type	block design

Design specifications | The functional localization experiment was divided into 8 blocks, including 4 tongue motor cortex and 4 laryngeal motor cortex localization blocks. The order of blocks was counterbalanced across participants. A resting period with the same duration followed each block. Each block contained 8 trials, each lasting for 2s.

Behavioral performance measures | During the experiment, participants needed to articulate the voiceless [t] (only tongue moved) or pronounce the voiced [a] (only laryngeal vocal fold vibrated).

Acquisition

Imaging type(s) | Functional and structural

Field strength | 3 Tesla

Sequence & imaging parameters | Structural image acquisition: T1-weighted anatomical image was acquired by a 3-Tesla Siemens Magnetom Trio scanner with a 20-channel head coil, using the magnetization-prepared rapid acquisition gradient echo (MPRAGE) sequence (TR = 2200 ms, TE = 3.49 ms, field of view = 256 mm, flip angle = 8°, spatial resolution = 1×1×1 mm).
Functional image acquisition: images were acquired by a continuous multiband-accelerated echo-planar imaging sequence (multiband factor = 4, 40 slices, TR = 640 ms, TE = 30 ms, flip angle = 25°, FOV = 192 mm, voxel size = 3 × 3 × 3 mm)

Area of acquisition | Whole brain

Diffusion MRI ☐ Used ☒ Not used

Preprocessing

Preprocessing software | fMRIprep (20.2.5)

Normalization | T1-weighted (T1w) anatomical images were corrected for intensity non-uniformity and skull-stripped with ANTs 2.3.3 4, and used as T1w-reference. Spatial normalization to standard space was performed through nonlinear registration with ANTs 2.3.3.

Normalization template | FSL's MNI ICBM 152 non-linear 6th Generation Asymmetric Average Brain Stereotaxic Registration Model

Noise and artifact removal | fMRIprep was used to correct for susceptibility distortions. Co-registrations of the BOLD to anatomical references were performed by ANTs 2.3.3. Head-motion parameters were estimated by FSL 5.0.9. Slice-timing correction was performed using AFNI 20160207. The BOLD time-series were resampled and realigned to the MNI152NLin6Asym standard space. AFNI 20220424 was applied for spatial smoothing (Gaussian filter FWHM = 6.0 mm) and scaling the BOLD time-series to a mean of 100 to derive percent signal change.

Volume censoring | fMRIprep was used to remove 4 TRs at the beginning of each block.

Statistical modeling & inference

Model type and settings | Generalized linear model (GLM) analyses were conducted at the individual level by AFNI. The predicted time course of BOLD activation was modeled as a "box-car" function convolved with the canonical hemodynamic response function at the subject level.

Effect(s) tested | Individual contrast maps were defined by comparing "AH" blocks to "D" blocks (dLMC: "AH" – "D", TMC: "D" – "AH") using paired t-test. Contrast maps were then subjected to group analysis where one-sample t-test (alternative hypothesis μ = 0) was used to find activated brain areas, which were thresholded by multiple comparison corrections (3dttest++ ClustSim program in AFNI 20220424, uncorrected $p < 0.001$, 10000 Monte Carlo simulations, smallest cluster size for "AH" – "D" = 239 voxels, "D" – "AH" = 164 voxels), and were masked by ROIs of bilateral precentral gyrus in automated anatomical labeling (AAL) templates.

Specify type of analysis: ☐ Whole brain ☒ ROI-based ☐ Both

Anatomical location(s) | bilateral precentral gyrus in automated anatomical labeling (AAL) templates

Statistic type for inference | voxel-wise

(See Eklund et al. 2016)

Correction | Monte Carlo

Models & analysis

n/a	Involved in the study
☒	☐ Functional and/or effective connectivity
☒	☐ Graph analysis
☒	☐ Multivariate modeling or predictive analysis
